# What is a B chromosome? Early definitions revisited

**DOI:** 10.1093/g3journal/jkae068

**Published:** 2024-04-16

**Authors:** Patrick M Ferree, Jelena Blagojević, Andreas Houben, Cesar Martins, Vladimir A Trifonov, Mladen Vujošević

**Affiliations:** Department of Natural Sciences, Pitzer College, Scripps College, Claremont, CA 91711, USA; Institute for Biological Research “Siniša Stanković” – National Institute for the Republic of Serbia, University of Belgrade, Belgrade 11060, Serbia; Department of Cytogenetics and Genome Analysis, Leibniz Institute of Plant Genetics and Crop Plant Research (IPK) Gatersleben, Seeland 06466, Germany; Institute of Bioscience at Botucatu, São Paulo State University (UNESP), Botucatu, SP 18618-689, Brazil; Research Institute for Limnology, University of Innsbruck, Mondsee A-5310, Austria; Institute for Biological Research “Siniša Stanković” – National Institute for the Republic of Serbia, University of Belgrade, Belgrade 11060, Serbia

**Keywords:** B chromosomes, genetic drive, selfish genetic elements

## Abstract

Since the discovery of B chromosomes, multiple different definitions of these selfish genetic elements have been put forth. We reconsidered early definitions in light of recently published studies. While there are many characteristics that vary among different B chromosomes, such as their evolutionary origins, size, segregation behaviors, gene content, and function, there is one defining trait of all B chromosomes: they are nonessential for the organism. The points raised here may be useful for framing future B chromosome studies and help guide the categorization of new chromosomal elements that are uncovered in genomic studies.

## Teaser

Reconsideration of how to define B chromosomes led to a central characteristic that unites these selfish genetic elements: they are non-essential for the organism. Other characteristics vary among different B chromosomes.

## Introduction

B chromosomes (Bs) are enigmatic heritable elements that have been identified in many fungal, plant, and animal species (Gutiérrez *et al.* 2023). The term “B chromosome” was coined in 1928 by Randolph to describe a chromosomal element he studied in maize (Randolph 1928). At that time, there was a central characteristic; because a B is supernumerary, or extra, to the standard “A” chromosomes of the genome, it must be nonessential. Since then, other descriptors have been raised. For example, it was suggested that a B is “a dispensable supernumerary chromosome that does not recombine with the A chromosomes and follows its own evolutionary pathway” (Beukeboom 1994). Other suggested criteria were that Bs should be morphologically distinct from and smaller than the A chromosomes, inherited in a non-Mendelian manner, impose fertility costs when present in high numbers, and have no genes with major effects (Jones and Rees 1982; Jones *et al.* 2008). This range of characteristics begs the question: are there any characteristics that unify all Bs? Having better clarity on this question would be advantageous for the scientific community; indeed, nearly every paper published on Bs includes a short description in its introduction.

We addressed this question by reconsidering several previous notions of what defines a B with respect to recent studies.

### All Bs are nonessential, but some can be helpful for the organism

Any chromosome that is required for viability or fertility should be deemed an A chromosome. However, certain Bs in plant–pathogenic fungi—also referred to as accessory chromosomes, supernumerary chromosomes, and lineage-specific or conditionally dispensable chromosomes (Soyer *et al.* 2018; Komluski *et al.* 2022)—can convey fitness advantages to those fungi, including the ability to infect a wider range of host plant species. These Bs are horizontally transferred between fungal species, although the mechanisms are unknown. Currently, the identities of the B-encoded virulence factors also remain unclear. While these Bs can provide benefits to fungi, they are not needed for the basic fungal life cycle or general pathogenesis. Thus, the benefits they convey do not violate their nonessentiality.

### Not all Bs drive (or do they?)

One of the most captivating characteristics of Bs is their ability to drive, i.e. transmit themselves at higher-than-Mendelian frequencies to progeny. B drive can occur during different developmental windows such as meiosis, the mitotic divisions preceding or following meiosis, and immediately after fertilization (Jones 2018). The cellular mechanisms vary widely, including *cis* actions like self-imposed B chromatid nondisjunction and preferential fertilization by B-carrying gametes (Birchler and Yang 2021) or *trans* effects like manipulation of reproductive development (Werren and Stouthamer 2003). However, some Bs, like one carried by the yellow-necked mouse (Karamysheva *et al.* 2021), may not possess drive (Vujošević *et al.* 2018). If true, then this B may persist through generations by stable mitotic and meiotic segregation, along with slight benefits conveyed to the organism. Alternatively, this B may drive but in a manner that is very subtle and thus hard to detect.

### Some B genes are functional (in B drive)

In recent years, there has been a surge in the use of genomic methods to identify B-encoded sequences. These studies have helped to understand how Bs originate and evolve. Additionally, uncovering B sequences begs the question: are any of them functional, despite prior expectations that they should not be? An obvious consideration is potential B gene functionality in drive. Several years ago, it was demonstrated that a gene expressed by a B in the jewel wasp, *Nasonia vitripennis*, is needed for B drive (Dalla Benetta *et al.* 2020). More drive genes will undoubtedly be uncovered for other B systems, and several studies are close. For example, B drive in maize involves B chromatid nondisjunction during the second pollen grain mitosis (Birchler and Yang 2021). When the tip of the B's long arm is translocated to an A chromosome, B drive persists, demonstrating the presence of a *trans*-acting factor expressed from the translocated region (Ward 1973). Sequencing efforts have delimited the causal gene(s) to 34 protein-coding candidates (Blavet *et al.* 2021). Thus, although most B genes are likely to be nonfunctional, some of them clearly have function.

### Once a B, not necessarily forever a B

Just as Bs partly or fully originate from A chromosomes (Camacho *et al.* 2000), it is also possible, even likely, that certain A chromosomes arose from Bs. Such may be the case for a B-like chromosome in the Pachón cavefish, *Astyanax mexicanus*. Different *Astyanax* species harbor large, mitotically unstable Bs that share an evolutionary origin in a common ancestor (Silva *et al.* 2021). Knockout of a B-encoded growth differentiation gene that is expressed in the male gonad tissue of *A. mexicanus* resulted in male-to-female sex reversal (Imarazene *et al.* 2021). Thus, it may be that the B in this species acquired a male sex-determining gene and, in doing so, became a sex chromosome. The related Bs in several other *Astyanax* species do not seem to be involved in male sex determination.

In another case, the avian germline-restricted chromosome (GRC), which is found in all examined passerine birds, may also have a B origin (Hansson 2019; Torgasheva *et al.* 2019). However, the invariable presence of the GRC in these birds argues strongly that this chromosome has some yet-to-be-defined functionality in the germline tissue. It is possible that, while certain chromosomes—like the B-like sex chromosome in cavefish and the passerine GRC—may have originated from Bs, once they become essential, such as through the acquisition of critical sex-determining or other needed genes, then they are no longer Bs and should not be referred to as such.

### Bs can, in certain cases, pair with A chromosomes

It is widely held that Bs do not pair and recombine with the A chromosomes (Jones *et al.* 2008). This pattern likely stems from the fact that Bs normally differ moderately to greatly in sequence composition from the A chromosomes. Indeed, it is reasonable to conclude that inappropriate B pairing with A chromosomes could lead to A chromatid missegregation and loss and, therefore, aneuploidy in gametes.

However, in a few cases, Bs have been observed to pair with certain A chromosomes during meiosis (cited in Chen *et al.* 1993). For example, some individuals of the wheatgrass, *Agropyron cristatum*, harbor 3 B copies. Karyotyping showed that, during the first meiotic division, 1 B copy paired at its distal end with an A chromosome, while the other 2 B copies paired together (Chen *et al.* 1993). Other, more complex pairing arrangements were also observed, including interactions among the A chromosome and all 3 Bs, simultaneously. A–B pairing was thought to arise from the similarity in subtelomeric sequences, and no A–B chiasmata (i.e. crossover structures) were observed. Thus, A–B recombination is not likely to occur in this case. However, such A–B pairing interactions do potentiate rare A–B exchange events, and, at minimum, they argue against complete B independence from the A chromosomes.

### Conclusion

**Fig. 1.**
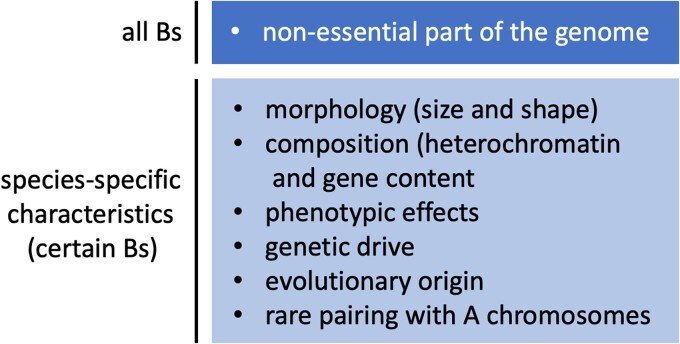
Characteristics that apply all B chromosomes versus those pertaining to certain B chromosomes.

Perhaps the only irrefutable characteristic of any B is nonessentiality (Fig. 1). This trait may be relatively straightforward to establish for newly discovered Bs because it only requires the demonstration of viable, fertile individuals with and without the B. Other B characteristics are conditional and/or species specific (Fig. 1). For example, Bs may or may not be beneficial to the organism; they rarely interact with the A chromosomes, but violations of this tendency are possible; they may drive in some cases but not always; and if they drive, they may express genes that facilitate this peculiar behavior or not. The same may also be true for other features that vary among Bs, such as their size, heterochromatin content, and evolutionary origins. Thus, great diversity exists regarding the properties of different Bs. For this reason, it seems reasonable to move toward a simpler definition of Bs—as nonessential extras. Such considerations may help frame future discussions of Bs in new manuscripts and provide some guidance for categorizing new chromosomal elements that turn up in discovery-based genomic studies.
